# Green Schoolyards as Outdoor Learning Environments: Barriers and Solutions as Experienced by Primary School Teachers

**DOI:** 10.3389/fpsyg.2019.02919

**Published:** 2020-01-09

**Authors:** Janke E. van Dijk-Wesselius, Agnes E. van den Berg, Jolanda Maas, Dieuwke Hovinga

**Affiliations:** ^1^Research Group Nature & Children’s Development, Thomas More Hogeschool, University of Applied Sciences Leiden, Leiden, Netherlands; ^2^Department of Experimental and Applied Psychology, Vrije Universiteit Amsterdam, Amsterdam, Netherlands; ^3^Department of Cultural Geography, University of Groningen, Groningen, Netherlands; ^4^Department of Clinical, Neuro & Developmental Psychology, Vrije Universiteit Amsterdam, Amsterdam, Netherlands

**Keywords:** collaborative action research, experiential learning, outdoor learning, reflective experiences schoolyard greening, teacher training

## Abstract

With a growing number of primary schools around the globe greening their schoolyards, opportunities arise to realize outdoor learning in natural areas on the school’s premises. Despite their promising potential, green schoolyards as outdoor learning environments remain mostly unintegrated in teachers’ educational practices. In the current study, teachers of five primary schools in Netherlands were followed for two consecutive years during a participatory action research project. Based on their experiences in this project, teachers identified barriers when integrating the green schoolyard as a learning environment and found practice-based solutions to overcome these barriers. Across schools, a total of 20 meetings were organized, with 75 teachers participating in the project. Results revealed four broad themes encompassing barriers and solutions. Teachers feel hindered by outdoor learning having no formal status in their current educational practice, experience barriers related to a lack of confidence in their own outdoor teaching expertise, find it difficult to get started, and experience barriers related to physical constraints. Teachers, professionals, and researchers together found solutions to overcome each specific barrier. These solutions can be translated to general recommendations: just do it, get educated and inspired, engage in real-life experiences, get an outdoor pedagogical mindset, and follow a tailored process. The findings can be used by primary schools and other institutions to develop interventions that support teachers to further integrate the green schoolyard as a learning environment.

## Introduction

Outdoor learning in natural areas can be an enrichment for children, enabling them to learn beyond the borders of their classroom, and has the potential to directly and indirectly strengthen primary schools’ educational practice (Rickinson et al., 2004; Blair, 2009; Wistoft, 2013; Goodall, 2016). Most literature regarding outdoor learning is concerned with activities in natural areas outside the school’s premises such as field trips, outdoor adventure activities, forest schools, school gardens, and nature education programs. Despite the promising potential of such extracurricular outdoor learning activities, teachers often feel hindered to facilitate and improve children’s access to these types of outdoor learning by factors related to transportation, curriculum requirements, and shortages of time and resources (Rickinson et al., 2004; Edwards-Jones et al., 2018). With a growing number of primary schools re-designing their schoolyards into green schoolyards with natural features such as grass, hills, trees, flowers, bushes, sand, and water, opportunities arise to realize more easy-to-accomplish outdoor learning activities in natural areas on the school’s own premises (Danks, 2010; Van Dijk-Wesselius et al., 2018). However, green schoolyards as learning environments remain mostly unintegrated in teachers’ educational practices. Amongst other reasons, this may be due to teachers’ unfamiliarity with outdoor learning and lack of hands-on experiences (Dyment, 2005; Maynard and Waters, 2007). As part of a two-year collaborative action research project, the current project examined the barriers teachers experience when they actually attempt to realize outdoor learning in the schoolyard, and what solutions they find to be supportive in overcoming these barriers.

### The Green Schoolyard as an Outdoor Learning Environment

Green schoolyards and other natural areas such as forests, parks, woodlands, and gardens afford a meaningful context for childhood education, as they provide children with numerous opportunities for both informal and formal learning experiences (Dyment, 2005; Auer, 2008; Ballantyne and Packer, 2009; Sahrakhiz et al., 2018). While playing in a green schoolyard, children are invited to handle, touch, smell, explore, and modify natural features with their entire body. These informal, child-initiated, embodied learning experiences can make important contributions to children’s emotional, cognitive, social, and physical development (Dyment and Bell, 2007; Kelz et al., 2013; Chawla and Nasar, 2015; Van Dijk-Wesselius et al., 2018).

Green schoolyards can also be used as an “outdoor classroom” for teaching regular classes in subjects such as reading, writing, mathematics, sciences, art, drama, and environmental education (Rickinson et al., 2004; Dyment, 2005). In this more formal approach to outdoor learning, learning comes alive through a kinesthetic, sensory, and experiential learning style (Lieberman and Hoody, 1998). Teachers become facilitators of learning and guide children through open and flexible real-life, bodily experiences that connect to a child’s abilities, needs, and interests (Harris, 2017). In outdoor learning, these hands-on experiences become the foundation for minds-on learning that extends beyond the formal curriculum (Lieberman and Hoody, 1998; Johnson, 2007).

A recurrent finding of research on the benefits of formal types of outdoor learning is that it raises enthusiasm, and increases vitality and motivation for learning (Rickinson et al., 2004; Wistoft, 2013; Waite et al., 2016). In addition, outdoor learning can reduce behavioral and concentration problems, particularly among children with difficult or mixed temperaments and children that are uninspired in the traditional classroom (Dyment, 2005; Blair, 2009; Fiskum and Jacobsen, 2012; Kuo et al., 2018; Largo-Wight et al., 2018). Other demonstrated advantages of outdoor learning include improved academic achievement, observational capability, and reasoning skills (Lieberman and Hoody, 1998; Ozer, 2007; Bell and Dyment, 2008; Blair, 2009; Becker et al., 2017; Browning and Rigolon, 2019), enhanced self-esteem, independence and feelings of responsibility (Rickinson et al., 2004; Ozer, 2007), improved interpersonal skills, cooperation and social cohesion (Ozer, 2007; Hartmeyer and Mygind, 2016; Waite et al., 2016), and multi-disciplinary learning across subjects (Harris, 2017).

### Barriers to Realizing Outdoor Learning in the Green Schoolyard

Despite the potential of green schoolyards as outdoor learning environments, outdoor learning tends to remain largely unrealized in educational practices (Skamp and Bergmann, 2001; Dyment, 2005; Maynard and Waters, 2007; Feille and Nettles, 2017). Surveys among staff and parents of pupils at primary schools in Canada (Dyment, 2005) and the United States (Feille and Nettles, 2017) show that only a small percentage of the teachers use green schoolyards as a learning environment. It is mostly used for physical education and science; most other subjects are rarely or never considered for teaching in the green schoolyard. Teachers express feeling hindered by a low confidence in their outdoor teaching expertise due to a lack of experience and knowledge. They report that curriculum requirements do not endorse or support outdoor learning and require the majority of teaching activities to be placed indoors. In addition, teachers indicate that broader issues within the educational practice and beyond, such as work pressure, overload in responsibilities, and a tiredness of educational changes hinders them from realizing outdoor learning in the green schoolyard.

More information on the barriers teachers experience when actually attempting to engage in outdoor education is provided by interviews amongst teachers from a primary school regarding their use of so called “learnscapes,” a concept related to green schoolyards that includes natural and built features designed to be used for outdoor learning activities (Skamp and Bergmann, 2001). Teachers, for instance, found management of children difficult, were uncertain on how to use and incorporate learnscapes, found planning of outdoor learning more complex, and struggled with outdoor learning not being a “real” thing. Furthermore, some teachers were timid about leaving the security of their classroom and the authors suggest that leaving the classroom requires a different “mindset.”

Several studies further reflect on outdoor teaching requiring a different mindset, and find that teachers feel hindered by an instrumental, indoor view on learning, and teaching (Dyment and Reid, 2005; Maynard and Waters, 2007; Waite, 2011; Passy, 2014). According to these authors, outdoor learning is considered to be more free and unstructured compared to indoor classroom learning, and is characterized by experiential and child-directed learning. Teachers can feel bound by an instrumental view on teaching in which they wish to stay in control and to be able to see all children at all times, and for instance, stick to predominantly teacher-directed lessons. It can be difficult for teachers to overcome this conflict within the realities of their ruling educational system. In this light, several studies stress the importance of a fundamental shift to recognize outdoor learning as a legitimate form of learning and an important part of core competencies of teachers (Dyment, 2005; Davies and Hamilton, 2018).

Altogether, findings from previous studies suggest that most teachers are familiar with an indoor pedagogical approach, and realizing outdoor learning in the green schoolyard requires them to discover the pedagogical opportunities of a new learning environment and overcome barriers related to their own didactical competence and demands of the curriculum. However, it remains unknown how teachers can overcome these barriers in their everyday educational practice.

### The Current Research

The current research was part of a larger collaborative action research project at five primary schools in The Netherlands. The project, called “becoming an outdoor teacher,” aimed to familiarize primary school teachers with using the green schoolyard as a learning environment and strengthen their didactical competence to realize and integrate outdoor learning in the curriculum. During the project, teachers gained hands-on experience of the barriers they face when trying to integrate the green schoolyard as a learning environment in their educational practice, and were stimulated to seek solutions to overcome these barriers and realize opportunities for outdoor learning at the green schoolyard. The current research aimed to gain more insight into these barriers and solutions, as experienced by teachers while experimenting with outdoor learning in the green schoolyard.

## Materials and Methods

### Context: Collaborative Action Research

The findings presented in this paper were collected in the context of a collaborative action research project. By maintaining the gestalt, the background and context of teachers’ daily practice, this type of project provides useful knowledge that has practical use (Khanlou and Peter, 2005). Collaborative action research is based on the assumption that new skills and knowledge in practices can be acquired when teachers systematically explore their own practice. In the collaborative approach used in the present study, researchers, and professionals support teachers in their systematic reflections and explorations. Through these collaborations, a community of practice emerges in which practice-based and practice-informed knowledge is developed together by teachers, professionals, and researchers. In this approach, the finding of solutions to overcome barriers is placed within the context of teachers’ hands-on experiences. This leads to the identification of solutions that are of direct relevance for teachers’ practices and can also be accumulated and transferred to other teachers, practices, and the development of theories (Ponte et al., 2004; Ponte, 2005).

**FIGURE 1:**
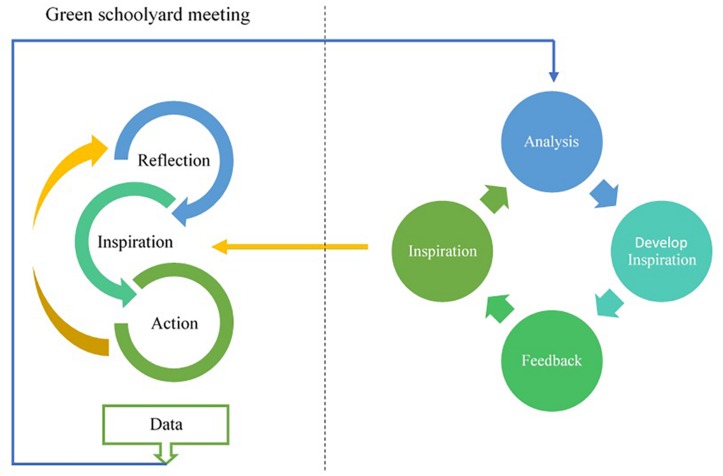
Collabrative action research design.

The collaborative action research was operationalized through so-called “green schoolyard meetings.” Several of these meetings were held during two consecutive years at each participating school. The cyclic process of collaborative action research is represented by a spiral of steps in each meeting. The meetings started with an evaluation phase. In this part, teachers reflected on the barriers and solutions they encounter in their experiences with outdoor teaching using an evaluation form and group discussion. This was followed by a phase that we labeled “inspiration moment,” consisting of exercises and other activities aimed to educate teachers. These inspiration moments were tailored to teachers’ specific needs. Finally, the last part of each meeting was the planning phase, in which teachers evaluated the inspiration moments and formulated a plan of action using an action planning form and group discussion. In the following meeting, the teachers reflected on the barriers and solutions they experienced while attempting to realize their planned actions, followed by an inspiration moment, and finally developing a new action plan. This ongoing cycle of evaluation, inspiration, and action is illustrated in the left part of Figure 1.

Throughout the project each individual teacher is in charge of their own goals, action planning, realization, and evaluation. Teachers directly benefit from their involvement in the project by professionalizing themselves as outdoor teachers. The role of the researchers was to facilitate the green schoolyard meetings and to support teachers in systematically evaluating barriers and solutions to realize their goals. The professionals had experience with outdoor learning in educational settings. Their role was to design and facilitate inspiration moments in collaboration with the researchers.

### Schools and Schoolyards

Six primary schools in western parts of The Netherlands participated in the project. A main selection criterion for inclusion of schools was that they should all have a green schoolyard upon entering the project and were located in urbanized areas with limited green play opportunities for children. Another criterion was that the green schoolyard should not yet be an evident part of teachers’ educational practice at the start of the project. School boards of schools that were potentially eligible for inclusion were approached directly by the research team. In a meeting with each potential school we discussed the onset of the project, required investment in time, and commitment of the school to start using the green schoolyard as a learning environment. Ultimately, six primary schools entered the project in two cohorts. Schools that declined to participate mainly declined due to a lack of time.

**FIGURE 2:**
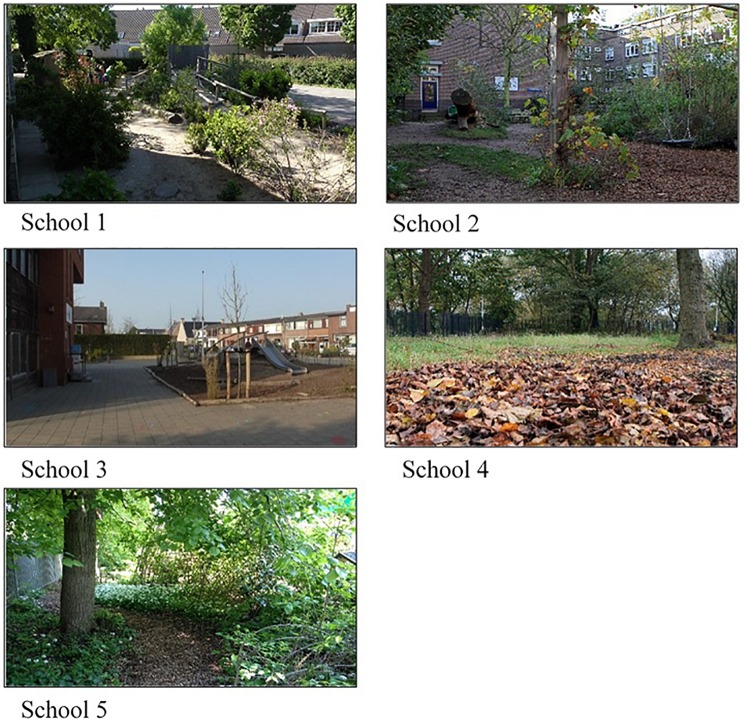
Impressions of the green schoolyards of each participating school.

Three schools started in the first cohort that ran from September 2014 till July 2016, and three schools started in the second cohort that ran from September 2015 till July 2017 (see Table 1). In the first cohort, one school quit the project after 1 month, for private reasons unrelated to the project. This school is not included in the present analysis, resulting in a final sample of five schools. Data from the remaining two schools in the first year of the first cohort were excluded from the present analyses, as they served to pilot test the materials. The two schools that remained in the first cohort included a school in an extremely urbanized area (>2500 addresses per square kilometer) and a school in a strongly urbanized area (1500–2500 addresses per square kilometer). Both schoolyards were greened for several years. The second cohort also included a school in an extremely urbanized area, as well as a school in a moderately urbanized area (1000–1500 addresses per square kilometer). In addition, the second cohort included a school for children with special education needs in a moderately urbanized area. The school in the extremely urbanized area had already had a green schoolyard for several years. The schoolyard in the moderately urbanized area had been greened for 1 year when the school entered the project. The school for children with special needs had a green area in the schoolyard that was destined to be further designed as a green schoolyard during the project. All schoolyards of the participating schools still had some paved parts with play equipment made of non-natural materials and green areas. The green areas in the schoolyards covered mostly features as grassy hills, bushes, trees, tunnels made of tree branches, loose tree branches, water parts, garden-like parts, and vegetable gardens. Figure 2 gives an impression of the green schoolyards.

**TABLE 1:** Total number of meetings and total number of teachers participating across meetings separate for each school and representation in the total sample in percentages.

	**Total number of meetings**	**Total number of participants across meetings**	**Total number of different teachers across meetings**
**Cohort 1**			
School 1^∗^	2	19(10.4%)	14(18.7%)
School 2^∗^	3	18(9.9%)	15(20.0%)
**Cohort 2**			
School 3	3	22(12.1%)	9(12.0%)
School 4	8	65(35.7%)	16(21.3%)
School 5	6	58(31.9%)	21(28.0%)
Total	20	182(100%)	75(100%)

*^∗^From the schools in cohort 1 only data of the meetings in the second year are included, data from the first year (five meetings) served as pilot data.*

### Meetings and Participants

At least six meetings were organized at each school across two consecutive years. Each meeting lasted for one and a half hours and was always held in the afternoon, after children were out of school. Due to issues non-related to the project school 3 in cohort 2 had to quit the project in February 2017, so at this school only three meetings were organized. Across the five schools, a total of 75 teachers (93.3% female) participated in a total of 20 meetings. The number of participants per meeting varied per school and per meeting, with a minimum of five in school 2 and a maximum of 13 in school 5 (see Table 1). More than two-thirds (69.7%) of the teachers participated in at least two meetings. At each school, teachers representing all grades, from children from the age of 4 till 11, participated. Answers of teachers were anonymized to ensure their privacy.

### Materials

During each meeting, teachers filled in two types of forms that asked them to reflect on their experiences with outdoor teaching (evaluation form) and the things that inspired them during the meeting (action planning form). Altogether, teachers filled in 182 evaluation forms and 182 action planning forms.

#### Evaluation Form

During the evaluation phase, to get insight into the barriers and solutions teachers experienced in their actions, teachers answered four open-ended questions on the evaluation form: (1) What defines your current experience with outdoor teaching?, (2) What did you enjoy?, (3) What barrier(s) did you experience?, (4) What supported you to overcome these barriers? These questions are based on previous studies using action research as a method to stimulate systematic reflection. This is a process that can allow teachers to increase awareness of their own experiences and stimulate a deeper form of learning beyond first impressions (Ponte, 2005). At the end of each meeting, each reflection form was photographed, so every teacher was able to keep their own reflection form.

#### Action Planning Form

During the action planning phase teachers filled in three open-ended questions on the action planning form: (1) What inspired you during this meeting?, (2) What implication does this has for your own educational practice?, (3) How are you going to realize this? The current paper only discusses answers to question 1.

### Procedure

During the project for each school one researcher was responsible for all communication and organization of the meetings. All researchers were trained by the leading researchers prior to the start of the project to ensure an adequate understanding of the design of the project and the use of the evaluation and planning form. Regular meetings between researchers were organized to discuss their experiences to increase the reliability and validity of findings. For instance, prior to the first meeting at a school, the researcher visited the school to get acquainted with it and to discuss the planning of meetings. The outcomes of these pre-focus meetings were discussed with all the researchers to ensure similarity in the onset of the projects on each school and minimize differences in data collection. Furthermore, after each meeting at each school researchers discussed their experiences and the analysis of barriers and solutions to increase triangulation of data analysis and the design of inspiration moments. After selecting salient barriers, the researcher together with the professional designed an inspiration moment. The proposed inspiration moment was discussed with the principal of each school to ensure that it focused on the most prominent barrier, and, if needed, the inspiration moment was further adapted to their needs. This process is illustrated in the left part of Figure 1.

The materials and procedure were pilot-tested with participants of the first cohort in the first year, and then adapted to better match the project’s intentions and reality of the primary schools’ daily practice. An important change concerned the implementation of inspiration moments in response to the observed need for education, in relation to the observation that teachers found it difficult to engage in actions due to a lack of familiarity with outdoor learning and ideas on how to get started.

### Data Analysis

Data were analyzed using qualitative content analysis (Mayring, 2000). Answers on each open question in the evaluation form and the first question in the action planning form were coded, categorized, and clustered into themes and subthemes by the researchers responsible for a school and the primary investigator. Themes and subthemes encompassed barriers and supportive aspects that teachers experience when facilitating outdoor learning. Analysis started with open, explorative coding of the original data based on similarities and relationships in the data. Answers to each separate question were read and primary codes were addressed in a few words. These codes were then compared in search for umbrella categories and clusters. Using inductive and deductive cycles, data was systematically assigned to these emerging codes, categories, and clusters. First, this procedure was followed for each question individually. Second, the categories and clusters were aggregated across questions. Subsequent data analysis by the primary investigator followed three phases, in which emerging themes and subthemes relating to barriers and solutions were increasingly aggregated from the individual team meetings to school- and supra-school level. After aggregating the inputs from individual researchers responsible for organizing meetings, the final analyses were completed by discussing the themes and subthemes with all researchers.

To increase consistency and saturation of the analyzed categories and clusters in themes and subthemes, a triangulation process was implemented in a few steps. First, separate for each school, after each meeting the responsible researchers transferred the analysis back to the school to ensure validity of the findings. Second, after analyzing the data from each meeting, researchers discussed ongoing analysis to compare categories and clusters between schools. Third, after completing the data collection and the subsequent meta-analysis across schools, the analysis was transferred back to all researchers and discussed in relation to the accurateness of aggregated themes and subthemes.

## Results

As illustrated in Table 2, barriers and solutions for using green schoolyards as outdoor learning environments can be summarized in four broad themes and subthemes. The most mentioned barriers relate to outdoor learning having no formal status in teachers’ educational practice (46.3%), followed by a lack of teachers’ confidence in their own outdoor teaching expertise (32.2%), physical constraints related to a lack of maintenance and weather conditions (13.0%), and finding it difficult to get started (8.5%). During the project, teachers, researchers, and professionals together found solutions to overcome each of these barriers. However, they found it relatively easy to find solutions to overcome a lack of formalization (64.8%) and to make it easier to get started (18.6%), while they found it relatively difficult to find solutions for strengthening teachers’ confidence (12.0%).

**TABLE 2:** Barriers and solutions experienced by teachers in number of times mentioned and percentages of total.

**No formal status outdoor learning**		**Lack of confidence in outdoor teaching skills**		**Difficult to get started**		**Physical constraints**	
**Barriers**							
Unfamiliarity and lack of inspiration	30(16.9%)	Fear of losing control	31(14.7%)	Difficult to get started	15(8.5%)	Lack of design and maintenance	13(7.3%)
Lack of time	28(15.8%)	Managing children’s behavior	26(17.5%)			Weather conditions	10(5.6%)
Lack of communal structure	24(13.6%)						
Total	82(46.3%)	Total	57(32.2%)	Total	15(8.5%)	Total	23(12.9%)
**Solutions**							
Inspiration moments to familiarize with outdoor learning	88(25.2%)	Teaching attitude	22(6.3%)	Decisive mind	26(7.5%)	Prevent child erosion	8(2.3%)
Inspiration by observing how children react to outdoor learning	34(9.7%)	Organization and rules	15(4.3%)	Step by step	17(4.9%)	Sunny weather	8(2.3%)
Inspiration through teamwork	33(9.5%)						
Conscious choice to devote time	25(7.2%)	Familiarize with outdoor learning	5(1.4%)	Pioneers	14(4.0%)		
Develop communal framework	23(6.6%)			Inspiration	8(2.3%)		
Teamwork	14(4.0%)						
Incorporate outdoor learning in the curriculum	9(2.6%)						
Total	226(64.8%)	Total	42(12.0%)	Total	65(18.7%)	Total	16(4.6%)

In the following sections, the barriers and solutions for each of the four themes will be discussed in further detail. Teamwork is found to be supportive across themes, and several aspects of teamwork will be discussed in relation to specific barriers and solutions.

### Theme 1: The Lack of a Formal Status of Outdoor Learning in Teachers’ Educational Practice

Teachers find it difficult that outdoor learning is not formalized in the current curriculum of their schools’ organization. This puts a challenge on teachers to formalize outdoor learning themselves, as they often they have no clear idea on what outdoor learning is and feel hindered by the demands of their existing curriculum. Within this theme, we distinguished three subthemes: unfamiliarity with the value and opportunities of outdoor learning in natural areas and lack of inspiration, lack of time, and lack of communal structure. For each barrier solutions were identified.

### Barrier: Unfamiliarity and a Lack of Inspiration

Teachers express the wish to meaningfully integrate outdoor learning within their educational practice but feel hindered by their own unfamiliarity with outdoor learning, and feel that their current didactical skills are inadequate to realize this. A teacher, for instance, expressed as a barrier: “*Both myself and the children are unfamiliar with the green schoolyard and outdoor learning. I need to learn so much myself before I can take the children outside. I have a fear of nature and no knowledge, so I am afraid that children will ask me questions that I cannot answer and I have no clue on what I am allowed and not allowed to do outside (2B1Z).”* Even if teachers already have undertaken some activities, they can still find it difficult to understand what they didactically can do with outdoor learning and how to meaningfully integrate it in their educational practice. As a teacher further exemplifies: *I started with enthusiasm to integrate the green schoolyard. Now I find it difficult, because I do not know exactly what I didactically can do with it (3B2Z)’* and another “*How can I integrate outdoors in my lessons? (3B2LA).”*

In response to an unfamiliarity with outdoor learning, some teachers express their need for inspiration and ideas. A teacher for instance wrote down “*I am a plant in need of nutrition (3B2Z).”* Further, after having done a first activity, some teachers feel hindered to continue with formalizing outdoor learning by not having new ideas and finding it difficult to keep generating new activities themselves. Teachers for instance literally wrote down as a barrier: “*What’s next? (5B2LA),”* and another “*To think up activities that are varied (5B5LA).”.* Related to this issue, some teachers express that it is difficult to “*To stay motivated (3B1LA)”* and “*To stay enthusiastic and motivated (3B2La).”*

### Solutions to Overcoming Unfamiliarity and a Lack of Inspiration

#### Inspiration Moments to Familiarize With the Value and Opportunities of Outdoor Learning

Teachers state that it was helpful during green schoolyard meetings to be inspired by an experienced outdoor teacher and to experience outdoor learning activities themselves. After meetings teachers wrote down as inspiring: “*The workshops outside (5B4I)”* and “*The green schoolyard meetings, that function as an example (2B6S).”* Actively participating in outdoor learning activities, such as short activities related to mathematics or language skills, supported teachers with familiarizing themselves with the concept of outdoor learning and lowered the threshold to actually start experimenting with outdoor learning in the green schoolyard themselves. A teacher, for example, described after an inspiration moment: “*I felt my shoulders relaxing, I definitely want to start doing it myself (2B3Z)”* and another, “*The tranquility I experienced by concentrated and with attention feeling the objects with my senses (3B2I).”* Teachers valued the simplicity of outdoor learning activities, and the suggestion to start with small and easy to carry out activities. Teachers wrote down as inspiring: “*The simple things you can do outside (3B3I)”* and “*Small things you are doing can already be big. Unconsciously there are a lot of learning opportunities (1B4I).”* Furthermore, teachers particularly valued activities that were accompanied by theoretical background on the value of outdoor learning. Teachers wrote down as inspiring: “*The activities with Marcel and the information on using your senses (4B5I)”* and “*The information on how a green learning environment inspires learning and fosters children’s ability to concentrate (1B4I)”* and “*Do not let children learn one-dimensional from books, but go outside to experience, move around, to make learning meaningful (3B1L).”* In addition, teachers felt inspired by opportunities to incorporate outdoor learning with existing subjects. A teacher for instance wrote down as inspiring: “*Develop your senses through small exercises in combination with vocabulary (1B1L).”*

After the meetings, we observed teachers integrating the inspiration in their own daily practice. For instance, teachers organized outdoor learning activities that provided children with experiences to use all their bodily senses, and guided them to further develop their observational skills. As teachers for instance reported on activities: “*A lesson on observing: Look at that tree. It’s color, it’s shape. Look again: tell me what else you see (4B6Z)”* and “*Senses, tasting, feeling, we practiced observing (4B6S).”* In addition, teachers connected exploring and observing natural features to subjects as mathematic and languages. Teachers wrote things down such as: “*Planting bulbs, measuring how deep. How does it feel? They emerge. Feel, smell, look at the earth, the clay and sand (3B2L)”* and “*Chestnuts, pine cones, shells as materials to practice mathematics (4B5Z).”*

#### Inspiration Through Teamwork

Teachers describe how they can inspire each other to facilitate outdoor learning in their educational practice through collaborating, sharing ideas, and experiences. Teachers, for instance, wrote down as supportive: “*Collaboration (4B7S)”* and “*Sharing ideas with a colleague (2B5S),”* and another teacher wrote down as inspirational: “*The stories and ideas from colleagues (4B4I).”* Realizing outdoor learning together can be a positive contribution to the team. A teacher, for instance, wrote down about her experiences: “*Joint responsibility for developing a focus for outdoor learning is an enrichment for the team (4B8Z).”*

#### Inspiration by Observing How Children React to Outdoor Learning

In all schools we observed that real-life experiences in teachers’ own daily practice are helpful for further familiarizing them with outdoor teaching. Teachers for instance described as supportive: “*Keep on experimenting (4B8S),”* and “*The day in which I tried out a few activities. Fun, surprising and informative results (5B4L).”* Across all schools and meetings, we observed how teachers are inspired in these real-life experiences by children’s reactions to outdoor learning. We observed an ongoing sense of joy and enthusiasm when teachers described their outdoor learning activities with children. For instance, the words “*Enthusiasm (4B1L)”* and that *’Children were having fun (5B4L)”* were mentioned frequently across all meetings by teachers when asked what they enjoy and what motivates them. Teachers for instance wrote down as inspiring: “*The children! By their enthusiasm (3B3I).”* In addition, teachers describe that they enjoyed observing children being wondered by natural elements, and how it opens up opportunities for learning. A teacher for instance wrote down as motivating: “*When children discovered something and are surprised about it (3B3L)”* and “*Children’s amazement about something (5B4L).”* Other teachers wrote down that they enjoyed: “*To observe how children were enjoying the mathematics assignment, without them really noticing that we were working on mathematics (4B8L)”* and “*Every child chooses for something else, I enjoy to see so many differences. It is really special to see that they choose something that really suits them (4B5L)”* and “*You are getting to know your children in a different way (5B3L).”*

Furthermore, some teachers value that outdoor learning activities can foster group dynamics, by stimulating social cohesion and collaboration amongst children. Teachers for instance wrote down that they enjoyed: “*To observe children collaborating in the schoolyard (5B5L)”* and “*A solid foundation for social cohesion in the group. Eating outside together: tranquility and social cohesion (5B3l)”* and “*Collaboration and discover each other’s strengths (and weaknesses) (4B4Z).”* Teachers also observed how outdoor learning fosters environmental awareness and enjoyed teaching children how to take care of the environment, respect nature, and overcome fears of nature. Teachers for instance wrote down that they enjoyed: “*Watering the plants (3B1L)”* and “*Children are getting more involved with nature. Searching for small insects, pretty flowers, and how do you take care of it (3B3L)”* and “*To observe a change within children. For instance a child that was scared at first for everything that was green and small (insects), and now behaves more comfortable and free and are more daring (4B8L).”* Lastly, teachers value the tranquility and space being outdoors literally can give, for instance to allow children to move around and relax: “*It meets children’s need to move around (4B7Z)”* and “*Children can calm down (5B1L).”*

These positive experiences with outdoor learning seem to enforce a motivation in teachers to further explore outdoor learning and their own capabilities as an outdoor teacher, and make time for outdoor learning. As they experience outdoor learning to be a valuable contribution, it becomes worthy to devote time to outdoor learning at the cost of something else. As a principal, for example, said during a meeting: “*It is the art of letting go. If something like this [ed. outdoor learning] comes in its place. At a certain point you have to do it (2B3Z).”* A teacher further explains: “*I experienced what it can bring, so it may cost time (2B4Z).”* For this particular teacher, lack of time was a main reason not to teach outdoors. However, after she experienced an outdoor learning activity, she was willing to invest time and even became a pioneer in her team.

### Barrier: Lack of Time

At the start of the first meeting, a few teachers simply wrote down the word “*Time”* as a barrier. We observed how important this was across the meetings, as teachers describe how their daily practice follows a tight and set schedule, in which outdoor learning literally has no place yet. Teachers for instance wrote down as barriers: “*I have a lot of ideas, but no time to give it a place in my daily practice (5B6Z)”* and “*I am looking forward to start, but I haven’t had the time to make a plan (5b2Z).”* Even if teachers have an idea for an outdoor learning activity, their tight schedule makes it difficult to find a moment to go outside. Teachers for instance reported as barriers: “*To schedule in time (4B8L)”* and “*To place my outdoor activity in my daily practice (3B1LA).”* The tight and set schedule of teachers is filled with a full educational program, with responsibilities and tasks that hinder teachers’ ability to invest time in outdoor learning. A teacher, for instance, wrote down as a barrier: “*It is difficult to make time besides all the other obligations, like CITO, monitoring learning outcomes, children’s behavior, meetings, etc. (4B8LA).”* As another teacher frames it: “*There is so much to do and so little time (5B4LA).”* A teacher further clarifies how in the ruling educational program finding time for outdoor learning is difficult, as it is becoming something additional, instead of an integrated and valued part of the curriculum. As she wrote down: “*It is difficult that there are only things being added to our work, but you also have to account for what you do. Barriers would be reduced if outdoor learning would be incorporated in our methods. Because: where do I find the time? Every additional thing that I do has to come from somewhere (2B2Z).”* Furthermore, within their full and tight daily practice, outdoor learning gets easily lost in other priorities. As teachers illustrate “*Our daily practice is too hectic at the moment, to prioritize outdoor learning (5B6LA)”* and “*Due to other priorities, I had insufficient time to practice with outdoor learning (5B1Z).”*

### Solutions to Overcoming a Lack of Time

#### Make a Conscious Decision to Devote Time to Establish Outdoor Learning

First, teachers mentioned it as helpful to consciously put outdoor learning activities on their schedule. Teachers for instance suggest to “*Schedule it in (2B4S)”* and “*Include it in the planning (5B6S)”* and “*Put what you intend to do on your schedule and execute” (4B8S).* Second, teachers suggest making time beforehand to prepare an outdoor learning activity. A teacher, for instance, wrote down: “*Preparation in terms of materials, etc.” (4B7S)* and another “*Preparations!!! (5B2S).”* Lastly, some teachers express the importance of creating a routine, and making it a habit to go outside. As teachers, for instance, wrote down: “*Repetition (5B3S)”* and “*Regularity (5B3S).”* Furthermore, pioneers in a team can support a conscious decision to devote time to integrate the green schoolyard as a learning environment, by taking responsibility for outdoor learning not getting lost in the hectic daily practice. In one school, for instance, a teacher wrote: “*There are two or three pioneers who actively manage the garden and consistently put it on the agenda, which keeps it alive (also in the autumn and winter) (4B8S).”* In addition, a few teachers suggest giving outdoor learning more priority by devoting time to the subject together as a team. A teacher for instance wrote down: “*The green schoolyard meetings (5B4S),”* and another wrote “*Put it on the agenda during team meetings (2B6S).”*

#### Incorporate Outdoor Learning in the Curriculum

A few teachers suggested searching for opportunities to connect outdoor learning to existing lessons and subjects to overcome a lack of time. A teacher for instance wrote: “*As an expansion after a method lesson on nature (3B1S)”* and another “*Relate the benefits from real-life learning outside to subject matters indoors (3B1S).”* In contrast, a few other teachers did not explicitly connect outdoor learning to a singular lesson, but focused on being aware of spontaneous moments during outdoor time to inspire outdoor learning. A teacher for instance suggested: “*Do not schedule an outdoor learning activity, but be aware for spontaneous moments (1B2S).”*

### Barrier: Lack of Communal Structure

Some teachers felt hindered by not knowing when they can use the green schoolyard. A teacher, for instance, wrote down as a barrier: “*For me it was unclear for a long time at what moment my class could go outside in the schoolyard (4B4Z)”* and another “*I could not do anything, my colleague cleared out the garden before I could start (4B8Z).”* Further, a lack of structure on how to use and share the green schoolyard for outdoor learning can lead to frustrations and uncertainty when teachers do go outside. A teacher wrote down as a barrier: “*Things that children built, were demolished later [ed. by other teachers and children] (4B4L)”* and another experienced “*It was overcrowded due to other classes that were outside (2B6LA).”* For other teachers, the lack of structure results in frustrations on sharing materials. As a teacher explains as a barrier: “*Keeping materials in line. I borrowed something to a colleague, and that is now in her classroom and I am standing with empty hands (5B4LA).”* Lastly, some teachers experience it as a barrier that there is no clear idea on what outdoor learning is and how it should be formalized within their school as an organization. A teacher for instance wrote down as a barrier: “*To me it is unclear what we want with it [red. outdoor learning]. It is a blank spot on the horizon, but how do we fill that spot and why in that manner? (5B1Z).”*

### Solutions to Overcoming a Lack of Structure

#### Teamwork

Teachers addressed a lack of structure by making rules on using the green schoolyard and organizing materials together as a team. They for instance wrote down: “*We made clear rules (4B4L)”* and “*Organize materials (4B5S).”* Teachers also found it helpful to exchange ideas with colleagues, a teacher for instance wrote down as supportive: “*Discuss with colleagues: Is a child always allowed to work outside? (5B6S).”*

#### Develop a Common Framework

In one particular school it was observed how a pioneer with a decisive mind sets in motion the development of a communal structure to establish outdoor learning in the green schoolyard. He wrote down as supportive: “*Lack of structure inspired me to develop a framework myself (5B3S).”* In one of the meetings he took the initiative to share his idea on working with so called “outdoor learning cards.” These are cards with outdoor learning assignments that are related to subjects in the existing curriculum. Assignments in particular stimulate real-life hands-on experiences in the green schoolyard, for instance related to mathematics, language, or creativity. During free hours, children can choose independently to go outside with a learning card together with another child.

Inspired by his idea, a group of colleagues took on the responsibility of further developing this framework and motivating colleagues to go outside and experiment with the outdoor learning cards. This seemed to work, as the team responded positively and found the framework supportive to go outside and start realizing outdoor learning. Teachers for instance wrote down as supportive: “*The format of our colleague (5B6S)”* and “*There is a clearer framework to work with (5B6Z).”* Teachers also reported enjoying noticing how outdoor learning becomes a more natural part of their daily practice by implementing the outdoor learning cards. A teacher for instance wrote down as motivating: “*To see what is all happening. And most of all. what we consider to be normal in outdoor learning (5B6L)”* and “*Children now can choose to do outdoor learning activities (5B6L).”* The development of the framework seems to provide a foundation to further integrate the green schoolyard as a learning environment.

### Theme 2: Lack of Confidence in One’s Own Outdoor Teaching Expertise

A recurrent theme concerns teachers reporting feelings of insecurity related to their own expertise as an outdoor teacher during outdoor learning activities. Within this theme, we distinguished the subthemes: fear of losing control, and difficulties in managing children’s behavior. To strengthen confidence in outdoor teaching expertise we observed three common solutions: Familiarize teachers with outdoor learning, organization and rules, and altering one’s teaching attitude.

### Barrier: Fear of Losing Control and Difficulties Managing Children’s Behavior

One aspect that teachers find difficult is how to cope with not being able to see every child at all times during outdoor learning activities in the green schoolyard, which makes it difficult to guard children’s safety and manage their behavior. Teachers for instance wrote down as barriers words such as: “*Overview (5B4LA)”* and “*Surveillance (4B6LA),”* and another teacher illustrates “*[red. children] out of your sight. Parents are worried about this (5B4LA).”* Teachers are used to an indoor setting in which the rules are clear; outdoors they are faced with a less structured learning environment. Not every teacher immediately feels competent to cope with this learning environment. A teacher for instance expressed as a barrier: “*Space and overview is sometimes difficult due to all the different areas (2B4lA)”* and another “*It is more difficult to address children (5B4LA).”* Teachers struggle with not knowing to which extent they can trust children’s behavior outside. As teachers illustrate as barriers: “*Measuring the size of the pond. Children are out of my sight, will they stay dry? (5B2L)“* and “*What can you expect from children (5B2LA)?”*

Some teachers struggle with safety and risk issues. They find it difficult to balance between warning and protecting children on one hand, and on the other hand allowing children the space to explore and take risks. A teacher for instance wrote down as a barrier: “*Warning for accidents is like a second nature. I need to learn how to restrain myself. As I often experience that it is not necessary (4B3LA)”* and another teacher admitted to finding it difficult: “*To see how children are taking risks, climbing in trees etc.…(4B3LA)”* and another “*Twigs are interesting and fun to play around with, but we also need to be attentive for risk (3B3LA).”* In addition, teachers experience that the level of independence you can trust a child with differs between children. As a teacher for instance wrote down as a barrier: “*Some children break the rules we have made, some children adhere to the rules (5B6LA).”*

Teachers also find it difficult to manage children’s behavior in a way so that all children will be engaged in the outdoor learning activity. Teachers attribute this problem partly to children being unfamiliar with outdoor learning. One teacher for instance wrote: “*I did not do it, my group was not ready yet (4B4Z).”* In addition, teachers themselves are unfamiliar with how to guide children during outdoor learning activities. A teacher for instance wrote down as a barrier: “*Guiding the children (5B6L)”* and another “*Management of the class. How can I stimulate free situations or invite children to behave free, quiet and motivated? (3B3L).”*

In particular at one school, teachers further reflected on a lack of confidence in their own expertise to generate and hold children’s attention during an outdoor learning activity. A teacher described as a barrier: “*Too many children under your guard, difficult to keep children involved (4B7LA)”* and another “*To go outside with the entire class, difficult to divide your attention (4B5LA).”* Furthermore, teachers experience difficulties in coping with children being attracted by the green schoolyard in a way that distracts them from the instructions or lesson they had scheduled as a teacher. A teacher, for example, wrote down as a barrier: “*To give instructions at the schoolyard. There are a lot of distractions for the children (4B7LA)”* and another “*Concentration of the children. This was sometimes diminished because they saw little insects or heard the sounds of for instance an ambulance or cars (4B8LA)”* This further shows how there can be a mismatch between the teachers’ intentions, and what triggers children during an outdoor learning activity or what children need to get engaged. As a teacher illustrates: “*It is difficult to stay together as a group. Children were looking for things that caught their interest (4B8LA)”* and another “*For some children an open assignment is too difficult. Running around, behaving crazy or really not being capable to make a choice (4B5LA).”* Furthermore, some teachers first consider it necessary to familiarize with their group indoors, before they can start with outdoor learning. A teacher for instance wrote down as a barrier: “*It is the beginning of the schoolyear, I am still unfamiliar with the children (4B4LA)”* and “*There are also three new children, who do not know each other”(4B4LA).*

### Solutions for Strengthening Confidence Expertise as an Outdoor Teacher

#### Familiarize With Outdoor Learning

Some teachers organized small step activities first that allowed themselves and the children to familiarize with outdoor learning in the green schoolyard. Teachers for instance did an exploratory walk with children around the schoolyard, let children draw their favorite place in the schoolyard, or had a lunch or reading moment outside. A teacher for instance wrote down as supportive: “*We took the period until the fall to familiarize children with the garden (4B4Z)”* and another “*With the children we did a tour in the garden, we explored what there is and how they can deal with the materials (4B4Z).”*

Furthermore, some teachers rely on repetition in order to let children adjust to outdoor learning and let it become ordinary: “*Assure regularity within the activities, so it becomes normal for the children (4B8S).”*

#### Organization and Rules

Other teachers try to overcome a fear of losing control by making rules and organizing outdoor learning. Teachers, for instance, discuss with children what is allowed and what is not during outdoor learning. As a teacher wrote: “*Discuss with children what surprised them in the schoolyard, but also about what you can and cannot do with loose branches (3B3S)”* and “*Talk about it with the children (4B4S).”* In addition, teachers find practical solutions to guard children’s safety by, for instance, assuring that younger children cannot open the fence. Furthermore, some teachers organize their instructions inside or find a paved, enclosed spot in the schoolyard to hold instructions. A teacher wrote down as supportive: “*Now and then I am in the ‘circle’ with my children, and I notice that I need this paved spot for instructions (3B3Z).”* Lastly, some teachers organize their outdoor learning in smaller groups of children, or only go outside if they have assistance from a colleague. A teacher for instance wrote down as supportive: “*Intern and teachers outside. One group can play, the other group is in the garden (4B5S)* and another *‘Smaller group, divide (2B4S).”’*

#### Altering One’s Teaching Attitude

Some teachers express how they learned to alter their own teaching attitude. They state that a key to cope with a fear of losing control is to trust on children’s independence and own sense of responsibility. A teacher, for instance, wrote down as supportive: “*Trust children that they can independently work outside on an assignment together (5B4S)”* and another “*Let children go, an trust on their own responsibility (4B6S).”* Teachers in this sense find a solution by increasing their own competence and allowing themselves to trust children and reflect on their own actions as a teacher to control and warn for risks. A teacher for example wrote down as a key: “*Be aware of your own actions, so you learn to diminish warning for risks (4B3S).”* During a meeting, a teacher further reflects on this issue of coping with risks by explaining: “*Most children know how far they want to go and stop for example with climbing a tree when they go to high. Risks are mostly in the environment, not in the child (4B4Z).”* Instead of focusing on their own fear to stay in control, these teachers focus on what is beneficial for children to learn outside in regards to risk taking and developing independence. In response, some teachers enjoy and feel motivation from experiencing that they indeed can trust children and observe how children are working on their own outside, as illustrated by remarks that: “*Children adhere to the rules (5B6L)”* and “C*hildren collect the materials on their own (5B6L).”*

To overcome barriers related to managing children’s behavior, some teachers reframed the question “what is distracting children?” to “what is attracting children outside?” They have an open and curious attitude, and become observant of children’s experiences in the green schoolyard. A teacher, for example, explains “*I have read with several children in the schoolyard and this helped me to further understand how children experience the outdoor environment. This supports me to further develop and experiment with outdoor education (4B8Z)”* and another teacher wrote down that she has been “*Observing how children experience the garden (4B3Z).”* By actively participating and playing with children, some teachers hope to attract children’s attention to an outdoor activity through their own enthusiasm and sense of wondering. As one teacher wrote: “*By being really enthusiast about something, for instance looking at a mushroom with amazement or a yellow leaf, you help the children to get engaged (4B5S)”* and “*Be enthusiastic yourself (4B7S)”* and “*Play along (4B5S).”* In particular, this holds for children who have more difficulty in getting engaged in an activity themselves. Furthermore, some teachers experience active participation as supportive to directly adjust their teaching style to children’s experiences: “*Actively participate myself. This allowed me to address children directly, stimulate them and resulted in interaction (4B3S).”*

### Theme 3: Difficult to Get Started

In particular in the beginning, when teachers have little to no experience with outdoor teaching, some teachers experience it as difficult to start with realizing outdoor learning in the green schoolyard. A teacher wrote down as a barrier: “*Getting started is the most difficult part (5B2LA)”* and another teacher described it as difficult “*To actually do it (5B3LA).”* Furthermore, a few teachers found it difficult to get started themselves, they wanted to wait and first experience how colleagues initiated outdoor learning activities. A teacher for instance wrote down: “*I hope to be caught by the enthusiasm of others – of pioneers (5B1Z)”* and another “*First see which way the wind blows (3B1Z);.”* In addition, some teachers feel too uninvolved with the concept of outdoor learning to stay engaged in the process of becoming an outdoor teacher. A teacher, for instance, wrote down as a barrier “*Outdoor learning is not on teachers’ mind in the higher grades (5B3LA)”* and another “*I cannot adequately empathize with this form of education, so I see almost no development [red. in my own activities] (3B3LA).”*

### Solutions to Getting Started

#### Decisive Mind

Teachers who feel hindered by outdoor learning not being formalized, express that a decisive mind supports them to overcome this barrier. Teachers for example report that “*Do it (5B2S)*” or “*Just start,”* and “*Instead of awaiting, make choices (5B2S)”* enabled them to go for it, to get engaged in first activities, and formalize outdoor learning themselves. A decisive mind is further characterized by “*Enthusiasm (5B2S),”* “*Feeling convinced (5B2S),”* and “*Perseverance (4B8S).”* This helps teachers to not give up after one activity, but instead continue to formalize outdoor learning despite of barriers they experience.

#### Step by Step

Teachers suggest taking a first small, demarked, and feasible step, and trust that step by step they will realize outdoor learning, as expressed by remarks to “*Keep it small (4B1S)”* and “*Trust, small steps also make a journey (5B1S).”* In addition, some teachers find it supportive to, as a first step, start indoors with a lesson that is related to the outdoor environment by bringing nature elements into their classroom. Teachers, for example, suggested, “*Walking stick bugs in the classroom (3B2S),”* “*Starting indoors (2B5S),”* and “*Only indoor sowing and planting (2B6Z).”*

#### Inspiration

Some teachers report inspiration with ideas on outdoor learning activities as a solution to overcome the hindrances of a lack of pre-structured lessons and methods for outdoor learning. “*A ready-to-use package with bulbs that a parent provided (3B1S)”* and “*Inspiration from other persons (3B3S)”* supported them to start with a first outdoor learning activity. In a later stage, a teacher mentions how you can get inspired by the environment to formalize outdoor learning, and another that it is important to free time to get inspired.

#### Pioneers

Previously we observed how in a particular school a pioneer set in chain a reaction of activities in other teachers to formalize outdoor learning. At other schools, teachers also described activities of “*A positive colleague who takes initiative (3B2S)”* and “*The spontaneity with which my colleague is going outside (4B8I)”* as a motivation to get started. The “*Chain reaction (5B2Lk)”* of outdoor learning activities, as one teacher described is, is not only supportive, but teachers also describe it as “*Catching (5B2Lk).”*

### Theme 4: Physical Constraints

Teachers report frustrations about the maintenance of the green space, in particular with the rapid deterioration of the green schoolyard. A teacher wrote down as a barrier: “*Quick deterioration of the green play hill (3B1LA)”* and another “*Rapid decay of the green schoolyard (3B3LA).”* Teachers experience it as difficult to protect the green schoolyard from children’s behavior. A teacher for instance wrote down: “*I brought a plastic white rose. This symbolizes how I love roses and enjoy looking at them. Our green schoolyard is being trampled and my rose withers (3B2Z).”* Furthermore, some teachers experience that the green schoolyard is not “green enough” for outdoor learning. A teacher, for instance, wrote down as a barrier: “*There are not enough green materials in our schoolyard (3B3LA).”*

Weather conditions are also mentioned as a physical barrier by teachers across all meetings. On most occasions, this concerns teachers who canceled an outdoor activity due to rainfall or stormy weather conditions. As a teacher for instance wrote down as a barrier: “*I brought a drawing of bad weather. This symbolizes the mathematics assignment I postponed. There was too much rain and wind (3B2Z).”* A few teachers mention specifically that certain seasons make outdoor learning more difficult; this was mentioned by teachers during the winter season. A teacher, for instance, wrote down as a barrier: “*The season impedes outdoor learning activities (4B6LA).”*

### Solutions to Overcome Physical Constraints

#### Preventing Child Erosion

To protect green areas against the so-called child-erosion, teachers find a practical solution. For instance, teachers placed: “*A red and white ribbon (3B2S)”* to protect flower bulbs. Furthermore, the team took upon initiatives to green their schoolyard with more natural materials, such as getting “*New plants through sponsoring (3B3S)”* and “*Bring materials myself, for instance 30 pineapples (3B3S).”* The team mentioned commitment to maintenance as important and enjoyed further designing their green schoolyard together.

#### Dealing With Weather Conditions

Whereas rainfall and stormy weather are mentioned as a barrier, sunny weather is considered inviting and supportive to go outside. Teachers for instance reported: “*Go outside, it is springtime! (5B6Z)”* and “*Nice weather for the garden (5B1L)”* and “*Schedule in outdoor lessons, but wait until the weather becomes a bit warmer (4B6S).”* Teachers who felt hindered by bad weather conditions, did not report on ways to overcome rainfall and stormy weathers. However, teachers do describe how experiences with seasonal influences in the green schoolyard inspired their outdoor learning activities. A teacher, for instance, observed with her children a chestnut tree across the seasons, as she wrote down “*Chestnut tree: we experienced all seasons! Bold, buds, leaves, autumn colors and chestnuts! (3B3Z)*” and “*Making fat balls for birds in January and February (4B6L).*” In another school the children made Christmas trees and decorations in the schoolyard with natural materials during the winter season. Still, fall and spring season seem easiest for teachers to experiment with outdoor learning. Heaps of leaves, chestnuts, and other natural materials in the fall, for instance, inspire creative learning activities, such as “*Crafting an autumn wreath with natural materials (4B6Z)*” and “*An Autumn craft corner (4B5Z).”* In springtime, teachers observe with children the emerging and blossoming nature and sow, care, and harvest kitchen gardens. As teachers, for example, wrote down: “*A free assignment: What has grown in the last week? (4B3Z)*” and “*Sowing and transpire. To observe the peas growing (5B6L).*” and “*Harvesting the grapes and eat them on a nice spot in the sun (2B4S).”*

## General Discussion

In this study we present data from a collaborative action research project called ‘Becoming an outdoor teacher’, in which we investigated barriers experienced by primary school teachers preventing them from facilitating outdoor learning in the green schoolyard, and solutions to overcome these barriers, across a period of two consecutive years. Results revealed four broad themes encompassing barriers and solutions. The first theme included three barriers related to outdoor learning having no formal status in teachers’ current educational practice: unfamiliarity and a lack of inspiration, lack of time, and lack of communal structure. The second theme included two, interconnected, barriers related to a lack of confidence of teachers in their own outdoor teaching expertise: fear of losing control, and difficulties managing children’s behavior. The third theme related to the barrier of finding it difficult to get started. The fourth theme related to physical constraints as posed by a lack of maintenance and weather conditions. These barriers are largely similar to those identified in previous studies by, for example, Dyment (2005), and Maynard and Waters (2007). However, a main contribution of the present research is that barriers were identified through a collaborative action approach, in which teachers, professionals, and researchers identified barriers through a process of systematic reflection on teachers’ real-life experiences. Moreover, the collaborative action approach challenged teachers, professionals, and researchers to come up with solutions to overcome barriers and realize outdoor learning in the green schoolyard. This provides meaningful data that are grounded in teachers’ daily educational practice.

To conquer the “daunting task” [as it was previously called by Dyment and Reid (2005)] of realizing outdoor learning in the green schoolyard we identified several solutions that could support teachers in overcoming the barriers related to each specific theme. With respect to the lack of formal status of outdoor learning (theme 1), as a solution to the barrier of unfamiliarity and a lack of inspirations, teachers found support in the organized inspirations moments, working together with colleagues, and engaging in real-life experiences and observing children’s positive reactions to outdoor learning. Teachers experienced that the barrier of a lack of time can be overcome by making a conscious decision to make time for outdoor learning, and to connect outdoor learning to existing lessons in the curriculum. To overcome a lack of communal structure, teachers also found teamwork helpful, as well as the bottom-up development of a common framework for outdoor learning. With respect to the lack of confidence of teachers in their own outdoor teaching experience (theme 2), teachers experienced that fear of losing control and difficulties managing children’s behavior can be overcome by familiarizing children with outdoor learning, making rules and organizing outdoor learning, and altering one’s own attitude as a teacher. To overcome difficulties in getting started (theme 3), a step by step approach, inspiration, a decisive spirit, and teamwork were found to be supportive. Finally, to deal with adverse physical conditions related to maintenance and weather (theme 4), teachers found support in practical solutions to prevent child erosion of the greenspace. Although teachers did not experience a solution to overcome rainfall and stormy weather conditions, they did find support in sunny weather, and found inspiration for outdoor learning in experiences with seasonal influences.

## General Recommendations

In addition to the specific barriers and solutions, some general recommendations for what is needed to realize outdoor learning in a green schoolyard can be derived from the present research.

### Just Do It

First, previous studies that theorized on what teachers need to realize outdoor learning mostly suggest the idea that teachers need to adopt a new pedagogical outdoor mindset (Dyment and Reid, 2005; Maynard and Waters, 2007; Waite, 2011; Passy, 2014). Although this sounds obvious, changing a mindset is difficult and costs time, which is scarce in current educational practices. Alternatively, the present research suggests that, when outdoor learning is yet another additional thing on the workload, the simple answer might be: just do it. There is a certain aspect of a decisive mind in some of the teachers. Despite all the barriers, despite the lack of time, despite the realities of their educational practice, they take a first step and go for it. Sometimes teachers were even surprised by their own actions. They did it, against their own odds. Scheduling it in, preparing, connecting outdoor learning to an existing subject, and collaborating with colleagues are some aspects that support this decisive mind. This mindset corresponds to a previous study in which ten primary school teachers in Scandinavia who gained some experience in the so called “udeskole” (teaching outside the classroom) were interviewed. Results showed that teaching outside can stimulate a feeling of regaining one’s professionalism (Barfod, 2017). However, the freedom and autonomy also create a double-edge sword as it puts a challenge on one’s professional judgment as a teacher. We also observed how teachers can enjoy using their skills and knowledge as a teacher to create outdoor learning, and at the same time can feel hindered by feelings of incompetence in regards to their unfamiliarity with outdoor learning and a lack of confidence in their outdoor teaching skill. In general, deciding that outdoor learning is a worthy part of one’s educational practice and just doing it can be a helpful strategy in realizing outdoor learning, but this also sets in motion a professional developmental process that brings to light doubts about one’s own competence and skills.

### Get Educated and Inspired

Second, we observed how inspiration moments and guided hands-on experiences can support teachers familiarizing themselves with the concept of outdoor learning, and opens up their awareness of opportunities to incorporate outdoor learning in the green schoolyard in their educational practice. In this sense, it seems of particular importance not to limit inspiration moments to ready-to -use lessons, but to combine theoretical background and real-life experiences aimed to stimulate a carry-over effect to teachers generating their own pedagogical ideas and meaningfully incorporate the green schoolyard as an outdoor learning environment in their educational practice. Only handing out concrete ideas for outdoor lessons can lead to a one-dimensional use of the green schoolyard and a failure to strengthen teachers’ professional judgment and competence. This can lead teachers to simply asking “what’s next,” and outdoor learning will risk to cease to exist when the inspiration flow stops, or all the lessons are carried out. This adds to a previous study that explored strategies that are effective in facilitating learning in a natural environment and stress the importance of teachers’ understanding the reason for visiting an outdoor location and having appropriate exercises to guide children in a meaningful learning process (Ballantyne and Packer, 2009). Without insight in the value and background of outdoor learning, time spent in the green schoolyard will be no more than a change of scenery instead of an enrichment of children’s learning experiences.

### Engage in Real-Life Experiences

Third, the importance of learning and inspiration goes hand in hand with the importance of real-life experiences in teachers’ educational practice and reflection on these experiences. Simply stated: teachers do not realize outdoor learning by staying indoors. They need to be stimulated to go outside, to experiment, to incorporate the green schoolyard as a learning environment through hands-on learning themselves. This builds upon previous research by Hickman and Stokes (2016) who evaluated outdoor leader education and training, and suggest the importance of reflecting on experiences in teachers’ daily practice to further professionalize and develop outdoor education skills. Experiencing outdoor learning for themselves meant barriers became more vivid compared to the barriers they had previously just imagined. Teachers sometimes experienced fears that they held that turned out to be different in reality, and vice versa. In addition, hands-on learning goes beyond acquiring physical and technical skills and supports the development of broader and holistic skills. In this there is a similarity between the characteristics of outdoor learning and what supports teachers to become an outdoor teacher. Through experiences outdoor education becomes alive, and teachers’ understanding and competence can be shaped and strengthened through practice. This is in line with previous research that discusses how outdoor learning can re-awaken joy in teachers (Waite, 2011).

### Get an Outdoor Pedagogical Mindset

Fourth, we observed that, although a controlling mindset based on fear of losing control and managing children’s behavior can be successful to a certain extent, it also entails a risk of a negative impact on the educational process. Stan and Humberstone (2011) observed in an ethnographic study teachers’ behavior during outdoor education and found that a controlling approach in order to manage risks during outdoor education limited learning opportunities for children. A different approach, in which teachers become observant to what attracts children in the green schoolyard, actively participated with the children, and aimed to understand the value of their (risky) behavior and guide learning activities, seemed to open up learning situations for the children. This builds upon previous studies, which suggest the need to develop a different attitude in which teachers loosen their indoor need for structure, and are open and curious to the opportunities of the unstructured green schoolyard (Dyment, 2005; Sahrakhiz, 2017a). Still, it remains somewhat unclear as to why some teachers embrace a more open mindset and other teachers hold on to indoor controlling strategies. One explanation could lie in teachers’ and schools’ vision on education and the school culture in this regard (Passy, 2014). Future research could extend our collaborative action research approach by observing and measuring the impact of teachers’ outdoor activities and behavior during these activities, and in reflections discuss these experiences in the context of their vision. This could further untangle what defines an outdoor pedagogical mindset, what supports teachers to develop this, and how their behavior can be grounded in a vision on (outdoor) learning.

### Follow a Tailored Process

Lastly, although most barriers are observed across schools, not every teacher has to experience or go through every barrier, and they may experience different barriers in different orders or phases. There can be differences between schools, but also differences within teachers at the same school. For instance, in one school the emphasis was on developing a communal structure for outdoor learning, while at another school managing children’s behavior was a major concern. In addition, while some teachers have ideas but find it difficult to make time for outdoor learning, others can struggle mostly with feelings of didactical incompetence. This reveals that becoming an outdoor teacher refers to a certain extent to a personal and organizational development. This implies that supporting teachers to facilitate outdoor learning in the green schoolyard requires a tailored process and there is no one-size-fits-all solution. This further builds on previous research that discusses differences between teachers in their willingness and capabilities to teach outdoors (Passy, 2014; Waite, 2011). Apparently to some teachers it becomes more natural to use the green schoolyard as a learning environment, while others are more hesitant to go outdoors. Interestingly, in a team it can become a strength that some teachers more naturally dare to get started and undertake outdoor learning activities in the green schoolyard. When sharing and making their activities visible to their colleagues, they can inspire and enthuse them to do the same. Furthermore, developing outdoor learning in the green schoolyard together as a team can be a valuable contribution to the school as an organization (Sahrakhiz, 2017b).

### Strengths and Limitations

To our knowledge, this is the first project that aimed to identify solutions that support teachers in overcoming barriers and realizing outdoor learning in the green schoolyard, and in which teachers were followed for two consecutive years. The collaborative action research design stimulated the development of hands-on knowledge of which teachers participating in the project directly benefited, and that can be extended to other primary schools on a national and international level. However, the research is not without its limitations.

First, primary schools participating in the project were open to devoting time to facilitate outdoor learning in the green schoolyard. This could have led to a self-selection bias, in which outcomes could be different in more reluctant schools. However, barriers observed are similar to those in previous studies in different countries. Furthermore, participating in the green schoolyard meetings was not without struggles. Despite a decision to participate in the project, teachers were often faced with other responsibilities that required their attention. In some occasions this led principals to decide to cancel meetings or to, in one situation, prematurely abandon the project. Although disappointing, this reflects a realistic situation of circumstances in which teachers need to establish outdoor learning.

Second, schools differ in the number of meetings and teachers participating in every meeting, and the designs of their schoolyards. In particular at one school, the type of greening and maintenance formed a barrier to realize outdoor learning. However, in qualitative research it is not about the quantity of measurements, but the content. Still, to account for differences between schools, we first aggregated findings within schools, and then triangulated our findings across schools. As shown, similar themes arose, but also differences between schools. These differences suggest a tailored process of becoming an outdoor teacher. Future research could devote attention to differences between schools and explore, for instance, whether these differences find their origin in the type of education, personality of teachers, or design of the schoolyard. For example, selection of schools based on systematic variations in school type and design or a procedure of co-analysis with teachers could account for these aspects.

Third, despite the fact that we observed teachers overcoming barriers and, in all schools, outdoor learning activities emerged, the project is not solely a success story. During and after the project barriers continued to exist, and teachers kept struggling with outdoor learning having no formal status and their own feelings of incompetence. However, a change has been set into motion and it is up to teachers to further rely on and strengthen their professional judgment. The supportive aspects found in the project can help teachers to continue their process. In addition, future research could support teachers by further investigating the impact of outdoor learning activities in the green schoolyard on children’s development and what constitutes a beneficial outdoor learning experience. As insight into the evidential value of outdoor learning can support teachers and institutions to acknowledge the green schoolyard as an outdoor learning environment and empower the formal status of outdoor learning.

## Conclusion

As a first project to explore what teachers need to facilitate outdoor learning in the green schoolyard, we hope to have set the stage for future research in unraveling the professional qualities of an outdoor teacher and the characteristics and value of outdoor learning in green schoolyards. Altogether, our research suggests that trusting on one’s professional judgment, taking the time and just doing it, getting educated and inspired, embracing an outdoor pedagogical mindset, engaging in real-life experiences, and reflecting on these experiences can support teachers to establish outdoor learning in the green schoolyard. Furthermore, our findings imply the importance of understanding why outdoor learning should be facilitated and stress the importance of teamwork.

## Data Availability Statement

The datasets generated for this study are available on request to the corresponding author.

## Ethics Statement

Ethical review and approval was not required for the study on human participants in accordance with the local legislation and institutional requirements. Written informed consent from the participants’ legal guardian/next of kin was not required to participate in this study in accordance with the national legislation and the institutional requirements.

## Author Contributions

JD-W and DH contributed to the conception and design of the study. JD-W organized the database, performed the qualitative content analysis, and wrote the first draft of the manuscript. DH and JM contributed to finalizing the data analysis and manuscript revision. AB revised the manuscript critically for important intellectual content. All authors approved the submitted version of the manuscript.

## Conflict of Interest

The authors declare that the research was conducted in the absence of any commercial or financial relationships that could be construed as a potential conflict of interest.

## References

[B1] Auer M. R. (2008). Sensory perception, rationalism and outdoor environmental education. *Int. Res. Geogr. Environ. Educ.* 17 6–12. 10.2167/irgee225.0

[B2] Ballantyne R. Packer J. (2009). Introducing a fifth pedagogy: experience-based strategies for facilitating learning in natural environments. *Environ. Educ. Res.* 15 243–262. 10.1080/13504620802711282

[B3] Barfod K. S. (2017). Maintaining mastery but feeling professionally isolated: experienced teachers’ perceptions of teaching outside the classroom. *J. Advent. Educ. Outdoor Learn.* 18 201–213. 10.1080/14729679.2017.1409643

[B4] Becker C. Lauterbach G. Spengler S. Dettweiler U. Mess F. (2017). Effects of regular classes in outdoor education settings: a systematic review on students’ learning, social and health dimensions. *Int. J. Environ. Res. Public Health* 14:E485. 10.3390/ijerph14050485 PMC5451936 28475167

[B5] Bell A. C. Dyment J. E. (2008). Grounds for health: the intersection of green school grounds and health-promoting schools. *Environ. Educ. Res.* 14 77–90. 10.1080/13504620701843426

[B6] Blair D. (2009). The child in the garden: an evaluative review of the benefits of school gardening. *J. Environ. Educ.* 40 15–38. 10.3200/JOEE.40.2.15-38

[B7] Browning M. H. Rigolon A. (2019). School green space and its impact on academic performance: a systematic literature review. *Int. J. Environ. Res. Public Health* 16:E429. 10.3390/ijerph16030429 30717301 PMC6388261

[B8] Chawla L. Nasar J. L. (2015). Benefits of nature contact for children. *CPL Bibliogr.* 30 433–452. 10.1177/0885412215595441

[B9] Danks S. G. (2010). *Asphalt to Ecosystems: Design Ideas for Schoolyard Transformation.* Oakland, CA: New Village Press.

[B10] Davies R. Hamilton P. (2018). Assessing learning in the early years’ outdoor classroom: examining challenges in practice. *Education* 46 117–129. 10.1080/03004279.2016.1194448

[B11] Dyment J. E. (2005). Green school grounds as sites for outdoor learning: barriers and opportunities. *Int. Res. Geogr. Environ. Educ.* 14 28–45. 10.1080/09500790508668328

[B12] Dyment J. E. Bell A. C. (2007). Grounds for movement: green school grounds as sites for promoting physical activity. *Health Educ. Res.* 23 952–962. 10.1093/her/cym059 17956885

[B13] Dyment J. E. Reid A. (2005). Breaking new ground? Reflections on greening school grounds as sites of ecological, pedagogical, and social transformation. *Can. J. Environ. Educ.* 10 286–301.

[B14] Edwards-Jones A. Waite S. Passy R. (2018). Falling into LINE: school strategies for overcoming challenges associated with learning in natural environments (LINE). *Education* 46 49–63. 10.1080/03004279.2016.1176066

[B15] Feille K. Nettles J. (2017). Permission as support: teacher perceptions of schoolyard pedagogy. *Electron. J. Sci. Educ.* 23 1–31.

[B16] Fiskum T. A. Jacobsen K. (2012). Individual differences and possible effects from outdoor education: long time and short time benefits. *World J. Educ.* 2 20–33.

[B17] Goodall J. S. (2016). Technology and school–home communication. *Int. J. Pedagog. Learn.* 11 118–131. 10.1080/22040552.2016.1227252

[B18] Harris F. (2017). The nature of learning at forest school: practitioners’ perspectives. *Education* 45 272–291. 10.1080/03004279.2015.1078833

[B19] Hartmeyer R. Mygind E. (2016). A retrospective study of social relations in a Danish primary school class taught in ‘u deskole’. *J. Advent. Educ. Outdoor Learn.* 16 78–89. 10.1080/14729679.2015.1086659

[B20] Hickman M. Stokes P. (2016). Beyond learning by doing: an exploration of critical incidents in outdoor leadership education. *J. Advent. Educ. Outdoor Learn.* 16 63–77. 10.1080/14729679.2015.1051564

[B21] Johnson P. (2007). Growing physical, social and cognitive capacity: engaging with natural environments. *Int. Educ. J.* 8 293–303.

[B22] Kelz C. Evans G. W. Röderer K. (2013). The restorative effects of redesigning the schoolyard. *Environ. Behav.* 47 119–139. 10.1177/0013916513510528

[B23] Khanlou N. Peter E. (2005). Participatory action research: considerations for ethical review. *Soc. Sci. Med.* 60 2333–2340. 10.1016/j.socscimed.2004.10.004 15748680

[B24] Kuo M. Browning M. H. Penner M. L. (2018). Do lessons in nature boost subsequent classroom engagement? Refueling students in flight. *Front. Psychol.* 8:2253. 10.3389/fpsyg.2017.02253 29354083 PMC5758746

[B25] Largo-Wight E. Guardino C. Wludyka P. S. Hall K. W. Wight J. T. Merten J. W. (2018). Nature contact at school: the impact of an outdoor classroom on children’s well-being. *Int. J. Environ. Health Res.* 28 653–666. 10.1080/09603123.2018.1502415 30047798

[B26] Lieberman G. A. Hoody L. L. (1998). *Closing the Achievement Gap: Using the Environment as an Integrating Context for Learning.* San Diego: State Education and Environment Roundtable.

[B27] Maynard T. Waters J. (2007). Learning in the outdoor environment: a missed opportunity? *Early Years* 27 255–265. 10.1080/09575140701594400

[B28] Mayring P. (2000). Qualitative content analysis. *Forum Qual. Soc. Res.* 1:20.

[B29] Ozer E. J. (2007). The effects of school gardens on students and schools: conceptualization and considerations for maximizing healthy development. *Health Educ. Behav.* 34 846–863. 10.1177/1090198106289002 16861584

[B30] Passy R. (2014). School gardens: teaching and learning outside the front door. *Education* 42 23–38. 10.1080/03004279.2011.636371

[B31] Ponte P. (2005). A Critically constructed concept of action research as a tool for the professional development of teachers. *J. Serv. Educ.* 31 273–296. 10.1080/13674580500200279

[B32] Ponte P. Ax J. Beijaard D. Wubbels T. (2004). Teachers’ development of professional knowledge through action research and the facilitation of this by teacher educators. *Teach. Teach. Educ.* 20 571–588. 10.1016/j.tate.2004.06.003

[B33] Rickinson M. Dillon J. Teamey K. Choi M. Y. Benefield P. (2004). *A Review of Research on Outdoor Learning*.

[B34] Sahrakhiz S. (2017a). Immediacy and distance in teacher talk—A comparative case study in German elementary- and outdoor school-teaching. *Cogent Educ.* 4:1291175 10.1080/2331186X.2017.1291175

[B35] Sahrakhiz S. (2017b). The ‘outdoor school’ as a school improvement process: empirical results from the perspective of teachers in Germany. *Education* 3-13 1–13. 10.1080/03004279.2017.1371202

[B36] Sahrakhiz S. Harring M. Witte M. D. (2018). Learning opportunities in the outdoor school–empirical findings on outdoor school in Germany from the children’s perspective. *J. Advent. Educ. Outdoor Learn.* 18 214–226. 10.1080/14729679.2017.1413404

[B37] Skamp K. Bergmann I. (2001). Facilitating learnscape development, maintenance and use: teachers’ perceptions and self-reported practices. *Environ. Educ. Res.* 7 333–358. 10.1080/13504620120081241

[B38] Stan I. Humberstone B. (2011). An ethnography of the outdoor classroom – how teachers manage risk in the outdoors. *Ethnogr. Educ.* 6 213–228. 10.1080/17457823.2011.587360

[B39] Van Dijk-Wesselius J. E. Maas J. Hovinga D. Van Vugt M. Van den Berg A. E. (2018). The impact of greening schoolyards on the appreciation, and physical, cognitive and social-emotional well-being of schoolchildren: a prospective intervention study. *Landsc. Urban Plan.* 180 15–26. 10.1016/j.landurbplan.2018.08.003

[B40] Waite S. (2011). Teaching and learning outside the classroom: personal values, alternative pedagogies and standards. *Education* 39 65–82. 10.1080/03004270903206141

[B41] Waite S. Bølling M. Bentsen P. (2016). Comparing apples and pears?: a conceptual framework for understanding forms of outdoor learning through comparison of English Forest Schools and Danish udeskole. *Environ. Educ. Res.* 22 868–892. 10.1080/13504622.2015.1075193

[B42] Wistoft K. (2013). The desire to learn as a kind of love: gardening, cooking, and passion in outdoor education. *J. Advent. Educ. Outdoor Learn.* 13 125–141. 10.1080/14729679.2012.738011

